# Local Acetaldehyde—An Essential Role in Alcohol-Related Upper Gastrointestinal Tract Carcinogenesis

**DOI:** 10.3390/cancers10010011

**Published:** 2018-01-05

**Authors:** Mikko T. Nieminen, Mikko Salaspuro

**Affiliations:** 1 Department of Oral and Maxillofacial Diseases, University of Helsinki, and Helsinki University Central Hospital, University of Helsinki, Biomedicum Helsinki P.O. Box 63, 00014 Helsinki, Finland; mikko.t.nieminen@helsinki.fi; 2 Research Unit on Acetaldehyde and Cancer, University of Helsinki, Biomedicum Helsinki P.O. Box 63, 00014 Helsinki, Finland

**Keywords:** acetaldehyde, ADH, alcohol, ALDH, ALDH2, cancer, ethanol, fermented food, tobacco, upper gastrointestinal tract

## Abstract

The resident microbiome plays a key role in exposure of the upper gastrointestinal (GI) tract mucosa to acetaldehyde (ACH), a carcinogenic metabolite of ethanol. Poor oral health is a significant risk factor for oral and esophageal carcinogenesis and is characterized by a dysbiotic microbiome. Dysbiosis leads to increased growth of opportunistic pathogens (such as *Candida* yeasts) and may cause an up to 100% increase in the local ACH production, which is further modified by organ-specific expression and gene polymorphisms of ethanol-metabolizing and ACH-metabolizing enzymes. A point mutation in the aldehyde dehydrogenase 2 gene has randomized millions of alcohol consumers to markedly increased local ACH exposure via saliva and gastric juice, which is associated with a manifold risk for upper GI tract cancers. This human cancer model proves conclusively the causal relationship between ACH and upper GI tract carcinogenesis and provides novel possibilities for the quantitative assessment of ACH carcinogenicity in the human oropharynx. ACH formed from ethanol present in “non-alcoholic” beverages, fermented food, or added during food preparation forms a significant epidemiologic bias in cancer epidemiology. The same also concerns “free” ACH present in mutagenic concentrations in multiple beverages and foodstuffs. Local exposure to ACH is cumulative and can be reduced markedly both at the population and individual level. At best, a person would never consume tobacco, alcohol, or both. However, even smoking cessation and moderation of alcohol consumption are associated with a marked decrease in local ACH exposure and cancer risk, especially among established risk groups.

## 1. Introduction

Alcohol is a major risk factor for upper gastrointestinal (GI) tract cancer and exhibits a dose-dependent effect on the incidence of oropharyngeal and esophageal squamous cell cancers [1]. A similar but weaker dose-risk relationship also exists with alcohol-related gastric cancer [1,2]. There is no evidence that the ethanol molecule itself is genotoxic, mutagenic, or carcinogenic [3,4]. However, its first metabolite, acetaldehyde (ACH), is a group 1 carcinogen to humans when associated with consumption of alcoholic beverages [5]. The International Agency for Research on Cancer (IARC’s) conclusion concerns metabolically formed ACH from ethanol and “free” ACH present in alcoholic beverages [5,6]. Therefore, knowledge of all factors regulating ACH concentration in the upper GI tract is of essential importance for cancer prevention. In other alcohol-related cancers, such as liver and female breast cancers, the carcinogenic mechanisms are different and by and large still hypothetical.

A point mutation in the aldehyde dehydrogenase 2 (*ALDH2*) gene results in deficient activity of the main ACH-metabolizing mitochondrial enzyme (ALDH2) and proves conclusively the causal relationship between local ACH exposure and upper GI tract cancers (Table 1; [7,8]). Bacteria and yeasts present in the normal upper GI tract microbiome play a major role in local ACH formation from ethanol present either in alcoholic beverages or food. Within seconds, alcohol ingestion results in a dose-dependent accumulation of ACH in saliva. The prominent instant increase of salivary ACH concentration is followed by a long-term phase, which lasts for as long as ethanol stays in the human body. Host metabolism of ethanol and ACH are controlled by organ-specific expression and gene polymorphisms of ethanol-metabolizing and ACH-metabolizing enzymes and contribute to microbial ACH formation. The most important exogenous sources for local ACH exposure are alcoholic beverages, tobacco, and alcohol-containing or ACH-containing (or both) “non-alcoholic” beverages and food. 

## 2. Acetaldehyde

### 2.1. Genotoxicity and Mutagenicity

In vitro and in vivo genotoxicity of ACH is generally accepted [3,5,9]. ACH fulfills four of the ten key characteristics that are commonly exhibited by established human carcinogens [10]. ACH is electrophilic and genotoxic, alters DNA repair, and induces oxidative stress. When administered in relatively high concentrations in vitro, ACH causes DNA-protein crosslinks, DNA-strand breaks, DNA adducts, sister chromatid exchanges, chromosomal aberrations, and micronuclei in eukaryotic cells. In a study using pig liver DNA, ACH concentrations ranging from 100 to 500 µM in the presence of polyamines resulted in an exponential increase of mutagenic ACH-DNA adduct (1,N^2^-propanodeoxyguanosine) formation [11]. These ACH levels are relevant for prevailing salivary ACH concentrations after alcohol ingestion [12,13]. On the other hand, increased polyamine synthesis is characteristic to regenerating oral and esophageal mucosa [14]. In 2015, the UK Committee on Mutagenicity concluded that the weight of evidence for the in vitro mutagenicity of ACH has been further strengthened, particularly with regard to generation of specific DNA adducts and induction of micronuclei in mammalian cells at ACH concentrations realistically achievable from consumption of alcoholic beverages [3].

Alcohol-treated *ALDH2*-knockout mice show increased levels of mutagenic ACH-DNA adducts in the tongue, submandibular glands, esophagus, stomach and liver [15,16,17]. Furthermore, Japanese alcoholics deficient in the ability to detoxify ACH (ALDH2 deficiency) combined with enhanced ability to produce ACH from ethanol (highly active alcohol dehydrogenase [ADH] enzyme) show markedly enhanced DNA damage characterized by elevated levels of N^2^-ethylidene-dG adducts in blood DNA [18].

A 12-fold to 16-fold increase in mutagenic ACH-DNA adducts (N^2^-ethylidene-dG) has been demonstrated in mouthwash samples of healthy human volunteers at 2 and 4 h after ingestion of a moderate dose of alcohol aimed to reach 0.7‰ blood alcohol concentration [19]. In an alcohol sipping model (diluted vodka at 5 min intervals) used in that study, salivary ACH concentration can be assumed to have been approximately 150 µM (maximum 260 µM) for the first 20 to 40 min followed by a rapid decrease to 20 to 30 µM for the remainder of the follow-up period (350 min) [8]. Mutagenic ACH concentration has been estimated to range from 40 to 100 µM [20]. 

The formation of mutagenic ACH-DNA adducts in oral mucosa has been confirmed in rhesus monkeys exposed to alcohol over their lifetimes [21]. In contrast to these findings, alcohol drinking did not appear to induce significantly increased levels of N^2^-ethylidene-dG adducts in blood white cells of human volunteers [22,23]. This discrepancy, however, is not surprising, since only very low ACH concentrations are found in hepatic venous blood of intoxicated non-alcoholic male Caucasians, and ACH levels are undetectable (<2 µM) in peripheral blood samples [24,25].

### 2.2. Carcinogenicity in Experimental Animals

There is sufficient evidence for the carcinogenicity of ACH in experimental animals [26]. In inhalation toxicity studies with rats, ACH vapor for up to 28 months produced adenocarcinomas of the olfactory epithelium at all exposure concentrations and squamous cell carcinomas of the respiratory epithelium at the two highest ACH levels [27,28]. Exposure to ACH vapor for 52 weeks produced nasal and laryngeal carcinomas in Syrian golden hamsters [29]. Without an obvious dose-response relationship, lifetime administration of ACH in drinking water to rats resulted in increased number of pancreatic adenomas, lymphomas, leukemias, uterine and mammary gland adenocarcinomas, and head osteosarcomas [30].

### 2.3. Carcinogenicity in Humans

Case reports and epidemiological studies on the carcinogenicity of ACH in humans without the presence of ethanol are very limited. Nine cases of malignant neoplasms were reported among an unspecified number of workers in an aldehyde factory in the German Democratic Republic between 1967 and 1972 [31]. Of the cancers found, five were bronchial tumors and two were carcinomas of the oral cavity. The authors stated that the relative frequencies of these tumors were higher than the expected frequency in the German Democratic Republic.

However, present epidemiologic and biochemical evidence on ALDH2-deficient individuals provides a unique and quantifiable ACH exposure model in humans, the kind of which is not available for any other of the group 1 carcinogens (Table 1, [7,8]). Nature has “randomized” millions of alcohol consumers to markedly elevated local ACH concentrations via saliva every time when drinking alcohol. The enhanced ACH exposure via saliva continues for as long as alcohol stays in blood circulation. Both of these factors can be quantified and linked to the risk for oropharyngeal cancer in relation to the amount of alcohol consumed daily. Most importantly, the model is not biased by confounding factors that hamper most epidemiological studies on alcohol-related cancer. 

#### 2.3.1. ALDH2-Deficiency as a Unique Human Cancer Model

A single point mutation in *ALDH2* gene results in deficient activity of the main ACH metabolizing low *K*_M_ mitochondrial enzyme (ALDH2). Consequently, ALDH2-deficient subjects are exposed in the presence of alcohol two to three times (via saliva) and five to six times (via gastric juice) higher ACH concentrations than those with the active ALDH2 enzyme [32,33,34,35,36]. Parallel to increased local ACH exposure, the risk of ALDH2-deficient alcohol drinkers for oral, pharyngeal, esophageal, and gastric cancer is manifold compared to alcohol-consuming individuals with the active ALDH2-enzyme [37,38,39,40,41,42,43]. The mutated gene (ALDH2*487Lys allele) is derived from the ancient Pai-Yuei tribe, which was widely distributed along the southeast coast of China up to Yunnan Province and the northern part of Southeast Asia 2000–3000 years ago [44]. Today its carrier frequency is about 600 million people of Eastern Asian descent [44,45]. Thus, ALDH2-deficiency is the most prevalent genetic health risk in the world, passing in frequency to that of familial hypercholesterolemia (FH). The prevalence of FH is 1:200–250, but in contrast ALDH2 deficiency is 1:13 [44,45,46].

#### 2.3.2. Quantitative Assessment of the Carcinogenicity of Acetaldehyde in Humans

The risk assessment for ACH has thus far relied on thresholds based on animal toxicology with lower one-sided confidence limit of the benchmark dose values (BMDL), typically ranging between 11 and 63 mg/kg bodyweight (bw)/day depending on species and endpoint [7]. However, the animal data is problematic due to poor study quality, problems in animal models, and relevance of the endpoint (cancer linkage) in translation to humans [7]. According to BMDL modeling, ALDH2-deficient heavy drinkers can be calculated to be exposed to a mean of 6.7 µg/kg bw/day of additional ACH via saliva compared to ALDH2-active individuals [7]. This associates with odds ratios of up to seven for esophageal and head and neck cancer [7]. These numbers derived from an ALDH2-deficient human cancer model suggest markedly lower BMDL values for ACH (<0.1 mg/kg bw/day) than the ones calculated from studies on experimental animals (11–63 mg/kg bw/day). Unfortunately, however, human data does not allow for a dose-response-modeling as none of the epidemiological studies provide absolute or extra risk data [7].

A simpler way is to calculate area under the curve (AUC) of local ACH exposure by multiplying salivary ACH concentration with exposure time (Table 2). Based on five studies with uniform results, salivary ACH concentration is a mean 1.1 mg/L higher in ALDH2-deficient subjects than in ALDH2-active subjects for as long as alcohol stays in the human body after its intake [7]. On the basis of the alcohol elimination rate (7 g/h), the increased exposure time to salivary ACH caused by the deficient ALDH2 enzyme is 283 min in moderate drinkers and 660 min in heavy drinkers. The total additional exposure of the oropharynx to ACH caused by ALDH2-deficiency is thus 311 mg/L/283 min in moderate drinkers and 726 mg/L/660 min in heavy drinkers. According to one meta-analysis and one well-performed study (total of 1189 cases and 3239 controls), ALDH2 deficiency is associated with a 1.68-fold to 2.61-fold oropharyngeal cancer risk in moderate drinkers and 3.57-fold to 7.28-fold risk in heavy drinkers [37,39]. The oropharyngeal cancer risk of ALDH2-deficient alcohol drinkers compared with that of ALDH2-active individuals thus appears to have a dose-dependent correlation with the additional local ACH exposure caused by the deficient ALDH2 enzyme [8].

Heavy alcohol drinking (ethanol ≥ 50 g/day) compared to non-drinkers and occasional drinkers is associated with a 5.13-fold risk for oropharyngeal cancer [1]. From 50 g of ethanol, the human body produces mathematically over 1 × 10^6^ µmoles of ACH. Hepatic ALDH enzymes, however, oxidize ACH formed from ethanol so effectively that after a moderate to high dose of alcohol (0.4–1.2 g/kg) blood ACH levels are not detectable in healthy individuals with the normal ALDH2 enzyme [24,25,53]. In ALDH2-deficient individuals, blood ACH levels are only slightly elevated (≤10 µM) [33,34,36]. In sharp contrast to the liver, the oral mucosa lacks low *K*_M_ ALDH enzymes and is thus unable to eliminate ACH formed from ethanol in the oral cavity [54]. The capacity of oral microbes to eliminate ACH is also low, and in this respect, there is no evidence for any differences between ALDH2-deficient and ALDH2-active individuals [55]. Therefore, alcohol drinking results in the accumulation of mutagenic concentrations of ACH in the saliva of both ALDH2-active and ALDH2-deficient individuals for as long as ethanol stays in the human body [12,13,33,34,35,36].

#### 2.3.3. Implications of the ALDH2 Cancer Model for Regulatory Authorities

There is no evidence that ACH derived from ethanol present sometimes in percentage concentrations in “non-alcoholic” beverages or food is less carcinogenic to the upper GI tract mucosa than ACH formed metabolically from the ethanol of official alcoholic beverages. This also concerns ACH found in mutagenic concentrations in fermented foodstuffs or when present in the product as a natural or added flavor [52,56]. There are no safe limits for very high (up to >1800 mg/L to >45,000 µM) ACH concentrations measured in some alcoholic beverages [57,58]. This can be explained at least in part due to the classification of ACH as a “generally recognized as safe” (GRAS) substrate validated by the Joint Food and Agriculture Organization of the United Nations (FAO) and World Health Organization (WHO) Expert Committee on Food Additives (JECFA) in 1998 and the Japan Flavour and Fragrance Materials Association (JFFMA) in 2015 [59,60]. Inappropriately, the decision tree for the safety assessment of flavoring substances applied by JECFA, JFFMA, the Flavour and Extract Manufactures Association (FEMA) Expert Panel, and the European Food Safety Authority (EFSA) is based on a misconception concerning ACH metabolism. The WHO model used by these authorities predicts that ACH undergoes complete metabolism to safe products [59,61]. This assumption, however, does not concern ACH, which accumulates in mutagenic concentrations in saliva in the presence of even very low ethanol concentrations (10 mM, 0.5‰) [12,13]. Furthermore, a peak (up to 1000 µM) in salivary ACH is seen instantly after sipping of alcoholic beverages containing high levels of ACH [13,36,62].

Genotoxic substances are generally not permitted for deliberate use in food production, since there is an increased risk for cancer even at low exposure levels. Therefore, controlling exposure levels to ALARA (EFSA) or ALARP (UK) (As Low as Reasonably Achievable or As Low as Reasonably Practicable, respectively) are generally accepted principles concerning genotoxic, mutagenic, and carcinogenic substances [10,63,64,65]. The scientific committee of EFSA has identified within its priority tasks the development of justifiable approaches for the assessment of risks from substances that are both genotoxic and carcinogenic [65]. To that aim, the ALDH2-deficiency model for local ACH exposure in the human oropharynx provides a novel tool for the quantitative reevaluation of ACH carcinogenicity in humans [7,8]. A key prerequisite for that approach will be that the amounts of ethanol and ACH in beverages and foodstuffs are made generally available for researchers, health care workers, regulatory authorities, and consumers.

## 3. Microbial Metabolism—A Major Source of Local Acetaldehyde in the Upper GI Tract

### 3.1. Salivary Acetaldehyde—Instant and Long-Term Exposure

**Figure 1:**
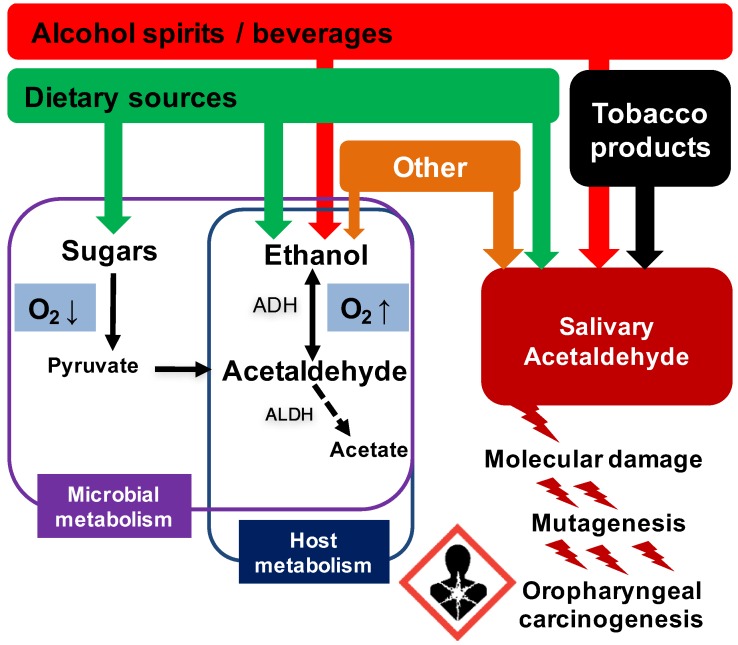
Schematic overview of the major factors contributing to salivary acetaldehyde (ACH) levels and therefore the local ACH exposure of oropharyngeal mucosa. ACH exposure leads to molecular changes and mutagenesis, including DNA-adduct formation, DNA-protein crosslinks, DNA strand breaks and chromosomal aberrations. Locally, these changes may lead to dysplasia and further into oropharyngeal cancer. Microbial and host metabolism share the ability to convert ethanol to ACH by the alcohol dehydrogenase enzyme (ADH). There is plethora of microbes that possess highly-active ADH enzymes. However, the subsequent conversion of ACH to less harmful substances seems to be very limited due to the lack of active aldehyde dehydrogenase (ALDH) enzymes in both microbial and oropharyngeal mucosal cells. In contrast to the human host, ethanol fermentation is a specific trait for microbes. In low oxygen tension, sugars derived from dietary sources, such as glucose and fructose, are converted into ethanol and ACH is formed as a byproduct. The possible contribution of fermentative pathways from glucose to local ACH production and further to ethanol has not been explored thus far in vivo. In addition to alcoholic beverages and spirits, multiple dietary constituents can contain ethanol, ACH, or both. There are also other sources of ethanol, such as hygiene products, including daily oral mouthwash use. In addition to alcohol, tobacco is the major contributor to salivary ACH.

Multiple factors contribute to salivary ACH levels (Figure 1). Normal human saliva does not contain measurable levels of ACH without the presence of ethanol or tobacco [12,50]. After sipping a small dose (5 mL) of 40% alcohol, ethanol is distributed to oral mucosal surfaces and saliva rapidly, resulting in an up to 900 mM (4.4%) salivary ethanol concentration at 30 s [13,48]. Instant microbial ACH formation from ethanol starts subsequently and continues for 5 to 10 min after each sip of alcohol (Table 2; [13]). Certain members of the normal oral microflora possess particularly high ADH activity and are able to dose-dependently produce ACH in vivo/in vitro in increasing ethanol concentrations from 11 to 1500 mM (0.5‰–7%) [12,66,67]. This indicates rather high *K*_M_ values for specific microbial ADHs and explains why in vivo salivary ACH concentrations are highest (up to 260 µM) immediately after sipping of alcohol [13]. 

One dose of alcohol (10 g ethanol) daily is associated with a significant relative risk (RR) of 1.17 (approximately 20% increase) for oropharyngeal cancer [49,68]. Alcohol intake in three sips at 5-min intervals is estimated to result in instant (mean approximately 115 mg/L/20 min) exposure of the oropharynx to ACH, which represents close to 70% of the total (168 mg/L/80 min) ACH exposure (Table 2, [13]). Long-term exposure (in this example approximately 53 mg/L/60 min) consists of ACH formed from ethanol that is diffused into saliva from blood and lasts for as long as ethanol is present in the human body [12]. High concentrations of “free” ACH present in some alcoholic beverages has been linked to the particularly high incidence of alcohol-related upper GI tract cancers [69,70,71]. This hypothesis is supported by biochemical findings showing that sipping of alcoholic beverages containing “free” ACH from 470 µM to over 15.5 mM results in a short, 1 to 2 min peak (up to ≥1 mM) in salivary ACH concentration [13,36,62]. Drinking habits, such as number of sips/dose and high ACH concentration in the alcoholic beverage consumed may thus have a significant effect on the exposure of oropharyngeal mucosa to ACH.

### 3.2. Oral and Esophageal Microbiome

As highlighted earlier, salivary ACH is mainly of microbial origin. There is a strong correlation between in vivo and in vitro salivary ACH production [12]. 4-methylpyrazole (4-MP) is an effective competitive inhibitor of the human ADH enzyme. Approximately 10 to 20 times higher 4-MP concentrations are necessary for 40 to 50% inhibition of in vitro microbial ACH production from ethanol in saliva and oral bacterial lysates than for equal inhibition of ethanol elimination in humans [12,34,67]. In ALDH2-active subjects, 4-MP (10–15 mg/kg) did not reduce salivary ACH levels but decreased the ethanol elimination rate by 46% [34]. This indicates the minor role of mucosal and glandular ADH and the major role of microbial metabolism in the regulation of salivary ACH concentration after alcohol intake. Furthermore, the use of chlorhexidine (CHX) antiseptic mouthwash for three days before intake of a moderate dose of alcohol decreased the levels of salivary ACH significantly (approximately 50%) in comparison with subjects that did not use CHX [12].

#### 3.2.1. Oral and Esophageal Microbiome in Health and Disease

Peak levels of ACH in saliva after alcohol ingestion can vary greatly between individuals (range 18–260 µM; [12,13]). These variations are due to compositional alterations in the oral microbiome and levels of ethanol in saliva after alcohol ingestion. In the oral cavity, the interindividual variations in microbial flora mainly occur at the species or strain level [72]. There are different intraoral habitats (e.g., lateral, dorsal tongue, and the gingival pockets) characterized by various environmental variables. Factors such as oxygen levels, nutrients, and type of surface structure (including squamous cell epithelium and the enamel of the tooth) affect the microbial community structure and functional outcomes. As elucidated by multiple microbiome studies, there is a highly diverse oral microbiome in health, including over 700 bacterial species [73]. The bacterial community structure is dominated by five phyla, including Firmicutes, Proteobacteria, Bacteroidetes, Actinobacteria, and Fusobacteria. *Streptococcus* is the leading genus in the bacterial spectrum, followed by *Prevotella*, *Veillonella*, *Neisseria*, and *Haemophilus.* The esophageal microbiome is a reflection of the oral microbial flora, broadly similar in composition to the oral microbiome [74]. Unfortunately, the rich fungal flora that interacts with bacteria in the upper GI tract is often overlooked in microbiome studies. As eukaryotes, fungi differ in multiple ways from prokaryotic bacteria. Fungal cells are larger in size and have specific cell-wall structures and advanced metabolic traits. The most common and well-studied are the *Candida* yeasts, especially *Candida albicans* species. There are also other fungal members, such as *Aspergillus*, *Malassezia*, and *Rhodotorula*, but their roles are widely unknown.

Poor oral health drives dysbiosis of the microbiome and is associated with dysplasia and carcinogenesis in the upper GI tract [75,76,77]. Microbiota isolated from premalignant and oral carcinoma sites have shown marked production of ACH in vitro, thus providing support for local ACH exposure [78]. In subjects with poor oral health, two-fold higher in vitro salivary ACH production from ethanol can be detected compared to individuals with good oral hygiene [79]. *Neisseria* and *Streptococcus* species not only dominate the bacterial microbiome in health but also in dysbiosis [80]. *Neisseria* and *Streptococcus* species can dose-dependently produce significant and mutagenic levels of ACH in vitro from ethanol [66,67]. Also, other species belonging to the core oral microbiome (such as *Rothia* and *Prevotella*) are able to produce marked quantities of ACH in vitro from ethanol [81]. The differences between species and genera are due to variations in bacterial ADH enzyme profiles. Pavlova et al. studied alcohol metabolism in streptococci and reported that many streptococcal species have highly active ADH but lack an active ALDH enzyme [55]. In certain species, ADH and ALDH enzymes are in the same enzyme complex. Similarly, no active ALDH enzyme has been found in *Neisseria* [67].

#### 3.2.2. *Candida* Yeasts—A Major Contributor to Local Alcohol Metabolism

In addition to bacterial counterparts, the fungal flora (*Candida* genus in particular) is a major contributor to local ACH exposure. The presence of *Candida* yeasts correlates significantly with high salivary ACH levels in vitro [82]. *Candida* yeasts are opportunistic organisms and thus their number increases in dysbiotic conditions (such as in individuals with poor oral hygiene or impaired immunity). Both *C. albicans* and non-*C. albicans* species, such as *Candida glabrata* and *Candida tropicalis*, can produce carcinogenic levels of ACH from ethanol in vitro [83,84]. To support the role of *Candida* yeasts in local ethanol metabolism and its linkage to malignant transformation, *C. albicans* ADH mRNA expression has been detected in oral dysplasia in vivo [85]. Furthermore, the virulence attributes of *C. albicans* and ACH production have been shown to correlate with the presence of oral cancer [86]. Chronic *Candida* infections have been linked to oral and esophageal carcinogenesis in susceptible individuals [87,88]. *Candida* yeasts are known for their fermentative traits, thus driving the metabolic flux from carbon sources (such as glucose and fructose) into ACH and ethanol (Figure 1). *Candida* yeasts can produce mutagenic levels of ACH from glucose in vitro [83,86]. Marttila et al. has shown that in low oxygen tension, conditions characteristic of multiple niches in the oral cavity, ACH levels produced in vitro from glucose by *Candida* can be orders of magnitude larger than when oxygen is present [78].

#### 3.2.3. Biofilm Lifestyle—The Preferred Mode of Microbial Growth in the GI Tract

Along the GI tract, the preferred lifestyle for microbes is the formation of biofilm communities. Biofilms are organized microbial communities attached to host surfaces and encased in microbially produced extracellular matrix. A well-known example of biofilms in the human body is dental plaque. In the biofilm mode of growth, microbial metabolism differs from its planktonic counterpart due to metabolic rewiring [89,90]. Fermentative growth is preferred especially in the deeper niches of biofilm structures, as oxygen is not as easily available as at the surface. Hypoxic conditions are common in multiple niches in the oral cavity, such as gingival pockets and the tongue. Due to these factors, the fermentative trait is preferred by yeasts and bacteria in the oral cavity and esophagus. Expression of genes related to ethanol metabolism is upregulated in *C. albicans* biofilms and high in vitro ACH production is associated with the biofilm lifestyle in *C. albicans* [91]. *C. albicans* may affect the site-specific bacterial flora within the biofilm and increase the number of anaerobic bacteria in the site in vitro [92]. This may increase the local ACH accumulation due to microbial fermentation. Unfortunately, past in vitro studies on microbial ACH production has mainly been performed using planktonic cells, which poorly simulates the growth and metabolism of various microbes in vivo.

#### 3.2.4. The Effect of Alcohol and Smoking on the Microbiome and Microbiome Ethanol Metabolism

Alcohol consumption, smoking, or both can alter the niche-specific microbiome composition and contribute to changes in biofilm structures and the biofilm metabolome [93,94]. Interestingly, in smokers the microbiota has a more potent ability to produce ACH from ethanol both in vitro and in vivo [50,79,95,96]. This may be due at least in part to selection of more ACH-tolerant species within the biofilms. In alcoholics, yeast overgrowth (in particular *Candida*) has been reported in vivo [97]. As shown in Russian and Japanese study populations, the gut microbiota in alcoholics differs from healthy controls with functional changes in microbial ethanol metabolism [98,99]. It remains to be studied whether similar changes also occur in the upper GI tract microflora. These compositional and functional changes, in combination with aberrant barrier function of the gut linked to alcohol use, can contribute to increased ACH exposure and provide a basis for local ACH-related carcinogenesis.

### 3.3. Gastric Microbiome

The stomach has long been considered a sterile environment due to low pH. However, novel studies have revealed that a core microbiome exists in a healthy acidic stomach as well [100,101,102]. The role of pH is critical in terms of balance in the gastric microbiome. A significant microbial overgrowth and dysbiosis exists in the stomach of patients suffering from hypochlorhydria or achlorhydria [103,104]. Hypochlorhydria or achlorhydria is usually due to atrophic gastritis or the use of acid-suppressive medication. Recently, Imhann et al. described a significant increase of genera ubiquitous in the oral microbiome (including *Streptococcus* and *Rothia*) in the gut of patients using acid-suppressive medication [105]. Under anaerobic conditions prevailing in some parts of a low-acid stomach and in the presence of glucose, bacterial colonization has been reported to result in an ethanol concentration up to 27 mM (1.3‰) and a maximum ACH concentration of 15.7 µM [106,107]. Väkeväinen et al. used culture-dependent techniques to identify the flora responsible for ACH production in hypochlorhydric patients [108]. Various anaerobic and aerobic bacteria as well as fungal species were identified [108]. The microbiome was rich in taxa and resembled the oral microbiome. The most numerous and prevalent species isolated were *Streptococcus*, *Stomatococcus*, *Neisseria*, and *Corynebacterium* species. High microbial ADH activities and low *K*_M_ values were determined for multiple species, thus supporting the potential for high local ACH production and exposure. More novel studies using culture-independent methods have shown the resemblance of microbial flora in various cancer-predisposing stages of stomach and gastric cancer with oral flora [109,110].

*Helicobacter pylori*, atrophic gastritis, alcohol consumption, tobacco smoking, acid-suppressive drugs, ALDH2-deficiency associated with alcohol consumption, pickled foods, fermented soy foods, and some fermented dairy products are risk factors for stomach cancer [1,2,38,43,111,112,113,114,115,116,117]. ACH is a common local denominator of all of these cancer risk factors or conditions. In the presence of alcohol, the intragastric ACH production in patients with hypochlorhydria or achlorhydric stomach secondary either to atrophic gastritis or the use of acid suppressive drugs is increased to mutagenic levels [32,107,108,118,119]. 

*H. pylori* is a core pathogen of the stomach. The presence of *H. pylori* leads to a decrease in the diversity of gastric flora. It is the most important etiologic factor for atrophic gastritis and gastric cancer, and is thus regarded as a group 1 human carcinogen by the IARC [120]. Many *H. pylori* strains possess an active ADH enzyme and can produce markedly high amounts of ACH from ethanol in vitro [121,122]. Under low oxygen tension, *H. pylori* might contribute to local ACH production through fermentation [123]. However, the in vivo ACH-related effects of *H. pylori* on metaplasia, dysplasia, and carcinogenesis are still unknown.

## 4. Host Metabolism of Ethanol and Acetaldehyde in the Upper GI Tract

Host metabolism of ethanol and ACH may contribute to microbial ACH formation from ethanol. These metabolic pathways are regulated differently by varying organ-specific expression of ADH, ALDH, and cytochrome P450 2E1 (CYP2E1) enzymes. In addition, gene polymorphisms have an effect on the activities of enzymes that metabolize ethanol and ACH.

### 4.1. Oral Mucosa and Salivary Glands

The human oral mucosa has minor ADH activity, 10% of that of the esophagus and ~6% of that in the liver [54,124]. Therefore, the role of mucosal ADH in ACH production from ethanol in saliva is presumably negligible compared with that of microbes (see Section 3.2). Buccal mucosa also expresses CYP2E1 [125]. However, the possible contribution of CYP2E1 to salivary ACH concentration in the presence of ethanol is not known. The oral mucosa lacks both cytoplasmic and mitochondrial low *K*_M_ ALDH enzymes [54]. This important characteristic apparently plays a decisive role in the accumulation of ACH in saliva after alcohol consumption.

There is strong evidence that after alcohol consumption the additional ACH is secreted in saliva in ALDH2-deficient individuals from the salivary glands. Sixty minutes after a moderate dose (0.5 g/kg) of alcohol, three Asian ALDH2-deficient volunteers (heterozygous for the mutant ALDH2*2 allele) had markedly elevated ACH levels (3.9–75.0 µM) in their sterile parotid gland saliva [33]. In sharp contrast, there was no measurable ACH in the parotid gland saliva from three ALDH2-active volunteers [33]. Furthermore, 4-MP, a powerful inhibitor of the human ADH enzyme, almost entirely prevented the ethanol-induced increase in salivary ACH in ALDH2-deficient subjects but not in those with the normal ALDH2 enzyme [34]. Finally, it was shown that ALDH2 deficiency has no effect on salivary ACH concentration without the presence of ethanol in the systemic blood circulation [48]. These findings indicate that ethanol is diffused to parotid glands from the blood circulation and is metabolized there to ACH. However, if the ALDH2 enzyme of the parotid glands is deficient, additional ACH is secreted to saliva. It remains to be established whether submandibular salivary glands play a similar role in additional ACH secretion in ALDH2-deficient individuals.

The ADH enzyme encoded by the ADH1C*1 allele metabolizes ethanol to ACH 2.5 times faster than that encoded by the ADH1C*2 allele. In heavy alcohol drinkers, this polymorphism is associated with increased risk for upper GI tract cancer [126]. In homozygotes, higher salivary ACH concentrations were observed following alcohol ingestion than those in volunteers heterozygous for *ADH1C* or homozygous for ADH1C*2 [127]. Among Japanese alcoholics, the ADH1B*2 allele encoding the fast ethanol-metabolizing ADH enzyme does not have significant effect on salivary ACH levels in the presence of ethanol as compared to those with the slow-metabolizing ADH1B*1/*1 genotype [35]. However, slow ADH may be associated with the prolonged presence of ethanol in blood and saliva and thus also with extended exposure time of the upper GI tract mucosa to microbial ACH [35].

### 4.2. Esophagus and Stomach

The regulation of ACH concentration in the esophagus in the presence of ethanol is much more complex and far less understood than that in the oral cavity. ADH activity of the esophageal mucosa is seven to twelve times higher than in the tongue and gingiva [54,128]. Furthermore, chronic alcohol consumption appears to induce CYP2E1 in the esophageal mucosa [129]. In contrast to the oropharynx, the esophageal mucosa also expresses some ALDH enzyme activity, which is ~3% of that in the liver [128]. The role of this ALDH enzyme in the local detoxification of ACH in the esophagus is not known. However, ALDH2 deficiency markedly and dose-dependently increases the risk for esophageal cancer among alcohol consumers when compared with ALDH2-active subjects, which indicates that the esophageal mucosa contributes significantly to the exposure of the esophagus to carcinogenic ACH [40].

Gastric mucosal ethanol and ACH-metabolizing enzymes are responsible for a significant gastric first-pass metabolism of alcohol. The stomach mucosa possesses ADH activity that represents one third of that in the esophagus and ~8% of that in the liver [130,131]. The gastric mucosa also contains ALDH enzymes with 2.2 times higher activity than in the esophagus and ~13% of that in the liver [130,131]. Furthermore, the gastric mucosa expresses some CYP2E1 activity [132]. However, the contribution of these various ethanol-metabolizing systems to the regulation of gastric juice ACH levels in the presence of alcohol is thus far unknown. In individuals with a normal acidic stomach and an active ALDH2 enzyme, intragastric alcohol infusion (0.5 g/kg) causes a slight increase in the ACH concentration of gastric juice from zero level to a mean peak of 10.4 µM [32]. However, in ALDH2-deficient subjects, a profound elevation to mutagenic ACH concentrations (mean peak 47.1 µM) was seen [32]. The highest intragastric ACH levels (mean 63.9 µM, range 32.0–96.7µM) were found in ALDH2-deficient subjects after seven-day treatment with an acid-suppressive drug (rabeprazole 10 mg b.i.d.) and intragastric alcohol infusion [32]. The alcohol-induced marked increase in gastric juice ACH concentrations of hypochlorhydric and achlorhydric patients, especially among ALDH2-deficient subjects, provides strong evidence for the local carcinogenic potential of ACH in gastric carcinogenesis. 

## 5. Exogenous Sources for Local Acetaldehyde Exposure

In alcoholic beverages, ethanol is a product of microbial fermentation. In the process, significant amounts of “free” ACH can accumulate depending on the microbial flora and fermentation process used in addition to distillation and preservation techniques. Certain alcohol spirits (such as fruit-based liqueur and spirits, grappa, sherry, and calvados) have been reported to contain extremely high levels of ACH (up to 1850 mg/L or approximately 45 mM) as a congener [57,58,62,71]. The high consumption rates of such spirits in certain Central European countries, such as France, Italy, and Hungary, may account for the unusually high local incidence of alcohol-related upper digestive tract cancers [69,71]. In addition, alcoholic beverages milder in terms of ethanol content (wine, beer, and cider) can contain levels markedly above the mutagenic (40–100 µM) limit of ACH [57,58].

There are also other routes for ACH exposure. ACH is added industrially to various daily products, including cosmetics and hygiene products. There has been discussion over the use of alcohol-containing mouthwash in daily dental hygiene routines. A large meta-analysis by Boffetta et al. reported that heavy use of alcohol-containing mouthwash is associated with a significant risk of oral and oropharyngeal cancers (ORs of 1.11 and 1.28, respectively) [133].

It is often forgotten and overlooked that diet can be a major local source for both ACH and ethanol. ACH can occur in food naturally, due to production processes, or added intentionally as a flavor compound. There is a long history of fermentation in food preservation. Interestingly, developments in food preservation techniques (i.e., emergence of refrigeration) has been shown to inversely correlate with the incidence of gastric cancer in developing countries [134,135]. However, there are still a plethora of undeveloped areas globally where conservative measures are used to preserve food. An example of such area is in Northwestern Iran where the use of certain milk-based products, cheeses, yogurt, and other domestic products have been found as independent risk factors for gastric cancer (Table 3, [117]). Although lactic acid is known as the major end product of fermentation, significant amounts of ethanol and ACH may also accumulate due to microbial fermentative processes. Ethanol concentration levels of 0.05 to 2.5% (10.5–525 mM) and even higher have been measured. Products mentioned in the literature include homemade mead and beer, kefir, kimchi, mursik milk, soy sauce, pickled food, and vinegar [56,136,137]. Mursik milk and pickled vegetables are examples of products associated with increased risk of esophageal cancer (Table 3).

Generally available commercial milk products (such as yogurt) and various fruits can also contain ACH in concentrations above its mutagenic limit (40–100 µM; Table 2) [52]. However, compared to alcohol-containing beverages and foodstuffs, the exposure time to ACH associated with the consumption of this type of product is much shorter (Table 2). In the safest-case scenario for one yogurt/day, the total daily exposure of oropharynx to ACH is 4.2% of that of one dose of alcohol. The corresponding percentage for one apple/day is only 0.5%. In the worst case-scenario, however, three yogurts (450 mL)/day with a particularly high ACH concentration (17.4 mg/L) would result in ACH exposure close to that of five cigarettes daily (Table 2). In a number of epidemiological studies, fermented food products have been established as risk factors for upper digestive tract cancers [56,112,114,140].

Alcohol is also used as part of cooking and added to certain sauces and dressings, including marinades, fondues, barbeque sauces, salads, and sushi. In contrast to common belief, alcohol does not totally evaporate during cooking. According to a study, 4 to 85% of alcohol is retained in food depending on the cooking procedure, with remaining ethanol concentrations of 0.06 to 4.21% (13–884 mM) after cooking [141]. As mentioned earlier, such concentrations of ethanol can lead to substantial ACH exposure locally.

ACH is one of the most abundant human carcinogens in tobacco [142,143]. Similarly, electronic cigarettes and various other smoking cessation products also contain ACH [144]. During smoking, salivary ACH level increases rapidly to a mean of 261 µM (11.5 mg/L) and remains elevated for as long as smoking continues [50]. After smoking, salivary ACH rapidly declines to basal zero level. The area under the salivary ACH curve for one cigarette is thus 58 mg/L/5 min and therefore the cumulative amount for three to five cigarettes ranges from 173 to 288 mg/L/15 min or 25 min/day. Individuals with no alcohol consumption history that smoke greater than three to five cigarettes/day have a 2.01-fold (OR) increased risk for head and neck cancer ([51], Table 2). Smoking independently increases the production of ACH from ethanol in saliva in vitro by 60–75%, and in combination with heavy drinking the increase in ACH production is up to 100% both in vitro and in vivo, especially in individuals with poor oral hygiene [50,79].

## 6. Preventive Actions—Detection of Risk Groups 

A key factor in cancer prevention is the identification of specific carcinogenic compounds and possible risk groups. ACH is a specific, local, and cumulative carcinogen. On the other hand, several environmental, genetic, disease-based, and even iatrogenic risk factors or conditions for upper GI tract cancer can be identified (Table 4). 

Alcohol and tobacco are overwhelmingly the most dominant risk factors for oropharyngeal and esophageal cancer. Their common carcinogenic denominator is ACH. Furthermore, alcohol consumption and smoking increase dose-dependently and synergistically both the exposure of upper GI tract mucosa to ACH and organ-specific cancer risk. 

However, with regard to alcohol and ACH-related upper digestive tract cancers, some important questions still remain unanswered. Microbial formation of ACH from ethanol present in “non-alcoholic” beverages, fermented food, or added to during food preparation forms a significant epidemiologic bias in upper GI tract carcinogenesis [8,56,114,117,141]. The same also concerns “free” ACH sometimes present in very high concentrations in commonly used alcoholic beverages and in mutagenic levels in some fermented food products or when used as a flavoring substance [52,56,57,58].

The most effective means to improve the poor prognosis of upper GI tract cancers is to improve recognition of risk conditions combined with early detection and treatment of precancerous conditions and individual health education based on updated information on hereditary, organ-specific and environmental risks. To that aim, key elements are regular checkups for good oral hygiene, appropriate *H. pylori* and atrophic gastritis diagnostics and recognition of increased gastric cancer risk associating with long-term use of drugs suppressing gastric acid secretion (Table 4). For example, it has been estimated that if moderate or heavy drinking ALDH2 heterozygotes were instead only light drinkers, 53% of esophageal squamous cell carcinomas might be prevented in the Japanese male population [45]. Therefore, screening of ALDH2 deficiency by flushing questionnaire or ethanol patch test combined with health education of those in the risk group has been recommended [45].

## 7. Minimization of Acetaldehyde Exposure

Considering the importance of cancer preventive measures, there are luckily ways to markedly reduce the exposure of upper GI tract mucosa to ACH at both population and individual level.

### 7.1. Population Level

Despite strong evidence on the local carcinogenicity of ACH in humans and the ubiquitous presence of ACH in our everyday life, the topic is poorly recognized among authorities and public health providers responsible for cancer prevention and food safety. Accordingly, outdated cancer risk assessments of ACH based on experimental animals need to be revised using uniform results obtained from genetic, epidemiologic, and biochemical studies on ALDH2-deficient alcohol consumers compared with ALDH2-active consumers [7,8]. Since there is no evidence that ACH exposure associated with the use of “non-alcoholic” beverages and food is less carcinogenic than ACH associated with the use of official alcoholic beverages, labeling of alcohol and ACH levels of commercially available alcoholic and “non-alcoholic” beverages and foodstuffs should be mandatory.

Currently, knowledge of the key role of local ACH in the pathogenesis of upper GI tract cancers is familiar only to a limited number of researchers working in the field. Therefore, updated evidence-based education should be provided widely for researchers, nutritionists, and consumers. In the near future, ALARA and ALARP should be overriding principles for the presence of ethanol and ACH in the production of alcoholic and “non-alcoholic” beverages and foodstuffs.

### 7.2. Individual Level

One standard dose of alcohol or three to five cigarettes daily significantly increases the risk for oropharyngeal cancer [49,51]. Therefore, it is vital to quit smoking, including electronic cigarettes, and to limit alcohol consumption to as low as reasonably achievable. If heavy drinkers were moderate alcohol consumers, the relative risk for oropharyngeal cancer would be 1.83 (instead of 5.13) and the corresponding value for esophageal cancer would be 2.23 (instead of 4.95) [1]. It should also be emphasized that individuals with normal ADH and ALDH2 enzyme profiles are not protected. For example, the relative risk of esophageal cancer in heavy drinkers (>120 g/day) and smokers (>30 g/day) is 149.1 as compared with never/light drinkers and smokers [148].

Maintenance of good oral hygiene is associated with an up to 100% decrease in microbial ACH production from ethanol both in vitro and in vivo [50,79,95]. Avoidance of beverages and foodstuffs containing ethanol, ACH, or both will hopefully be possible when this information is made available to consumers.

Exposure of the upper GI tract mucosa to ACH via saliva or gastric juice after alcohol administration can be markedly decreased by using special drug formulations of slow-release L-cysteine. L-cysteine is a semi-essential, natural, and safe sulfur-containing amino acid that inactivates ACH by binding to it non-enzymatically. The compound formed, 2-methyl-thiazolidine-4-carboxylic acid (MTCA), is excreted in the feces or metabolized to safe substances in the liver. A buccal tablet containing 100 mg of slow-release L-cysteine eliminated 60% of ACH in saliva for 5.3 h after ingestion of a moderate dose (0.8 g/kg) of alcohol [149]. A lozenge containing 5 mg or 2.5 mg of L-cysteine inactivates during smoking 100% and 96% of salivary ACH, respectively [150].

After intragastric alcohol installation in patients with atrophic gastritis, 60 to 70% of gastric juice ACH is eliminated by ingestion of two slow-release L-cysteine (100 mg) capsules [118,151]. A similar inactivation of ACH after alcohol administration can be found also both in ALDH2-active and ALDH2-deficient individuals treated for one week with a proton pomp inhibitor (rabeprazole 10 mg *b.i.d.*) [32].

## 8. Conclusions and Future Directions

Local microbial, mucosal, and salivary gland ACH production in the oral cavity as well as “free” ACH in multiple beverages and foodstuffs are considered to play important roles in the carcinogenesis of alcohol-related upper GI tract cancers.

Microbial formation of ACH in mutagenic concentrations starts in saliva and in the gastric juice of the achlorhydric stomach instantly after alcohol ingestion and continues for as long as ethanol is present in the human body. The key role of the oral microbiome, bacteria in particular, has already been well established in this process. The role of the fungal flora composition in the upper GI tract has started to gain increased attention. In terms of ethanol and ACH metabolism, more effort is needed to further characterize the fungal-bacterial metabolic interactions. Current evidence also underlines the importance of microbial biofilms along the upper GI tract, which are complex high-order communities with unique metabolic traits in comparison to planktonic microbes. 

A point mutation in the *ALDH2* gene provides a unique human cancer model for local ACH exposure, the kind of which is not available for any other of the group 1 human carcinogens. Accordingly, previous animal toxicology-based risk assessments of ACH need to be revised using updated evidence from genetic-epidemiologic and genetic-biochemical studies on ALDH2-deficient subjects compared with ALDH2-active subjects.

Smoking cessation and abstinence or moderation of alcohol consumption are key factors in the prevention oropharyngeal and esophageal cancers. There is currently no evidence that ethanol and ACH present in “non-alcoholic” beverages and food is less carcinogenic than ACH associated with consumption of official alcoholic beverages. The same also concerns ACH present in very high concentrations in some alcoholic beverages. Therefore, mandatory labeling of ethanol and ACH concentrations of commercially available beverages and foodstuffs would be of vital importance for researchers, health care workers, nutritionists, and consumers. 

## Figures and Tables

**Table 1:** Main characteristics of the gene mutation-based human model for the quantitative assessment of acetaldehyde (ACH) carcinogenicity in alcohol-consuming aldehyde dehydrogenase (ALDH) 2-deficient subjects compared to ALDH2-active subjects [7,8].

➢ The ethanol molecule is neither genotoxic, mutagenic, nor carcinogenic ➢ ACH associated with alcohol consumption is a Group 1 human carcinogen ➢ ACH does not exist in saliva without the presence of ethanol or tobacco ➢ ACH accumulates after alcohol intake in about 2 times higher concentrations in saliva in ALDH2-deficients than in ALDH2-actives ➢ The oropharynx and esophagus (?) as ideal target organs for quantification of ACH-related cancer risk in humans ▪ In sharp contrast to the liver, the oral mucosa lacks low-*K*_M_ ALDH enzymes and is thus unable to eliminate acetaldehyde formed from ethanol ▪ The expression of ALDH enzymes is low in the esophagus, but their role in the regulation of local ACH levels is still unknown ▪ The oral microflora has low or zero capacity to eliminate ACH ▪ Adequate data on salivary ACH in ALDH2-deficient subjects vs. ALDH2-active subjects; equivalent data on ACH of the mucosal surface of the esophagus is still unavailable ▪ Adequate epidemiological data on the role of ALDH2 polymorphisms on the risk for oropharyngeal cancer among never, moderate, and heavy drinkers ➢ No differences between ALDH2-deficient subjects and ALDH2-active subjects in confounding factors hampering most epidemiological studies on alcohol-related cancer ▪ Smoking, diet, consumption of different beverages, varying drinking habits, underreporting, oral hygiene levels, human papilloma virus (HPV), body mass index (BMI)

**Table 2:** Effect of ALDH2 deficiency, alcohol consumption, smoking, and certain foodstuffs on the quantitative exposure of oropharynx to acetaldehyde (ACH) via saliva in relation to the risk for oropharyngeal cancer. Calculations are based on selected examples as indicated in footers.

Exposure Model	Salivary ACH Concentration (mg/L; µM)	Exposure Time (min)	ACH Exposure (mg/L × min)/day	OR/RR for Oropharyngeal Cancer
**ALDH2-Deficiency Model**				
- 3 doses (33 g ethanol)/day	1.1; 25 ^1^	283 ^2^	311 ^1^	1.68–2.61 ^3^
- 7 doses (77 g ethanol)/day	1.1; 25 ^1^	660 ^2^	726 ^1^	3.57–7.28 ^3^
**1 Dose 40% Alcohol, no ACH ^4^**				
- Instant				
3 × first 5 min	6.2; 150	15	93	
1 × next 5 min	4.4; 100	5	22	
instant total			115	1.29 ^5^
- Long-term				
20–80 min	0.88; 20	60	53	
- Total				
0–80 min		80	168	
**Smoking ^6^**				
- 1 cigarette	11.5; 261	5	58	
- 3–5 cigarettes	11.5; 261	15–25	173–288	2.01^7^
**Yogurt**				
- Safest-case scenario ^8^	2.4; 55	3	7	?
- Worst-case scenario ^9^	17.4; 395	3 × 5	261	
**Apple**				
- Safest-case scenario ^10^	0.3; 7	3	0.9	?
- Worst-case scenario ^11^	2.4; 55	3 x 5	36	

^1^ Difference in salivary ACH concentration (column 2) or in ACH exposure (column 4) (ALDH2-deficient subjects—ALDH2-active subjects) [7]; ^2^ Based on the normal elimination rate of ethanol (7 g/h) [47]; ^3^ Odds ratios (ORs) for oropharyngeal cancer, ALDH2-deficient subjects compared to ALDH2-active subjects [37,39]; ^4^ One dose of alcohol is assumed to be ingested in three sips at 5-minute intervals staying each in the mouth for about 5 s before swallowing. Adapted from [12,13,48]; ^5^ Relative risk (RR) of oropharyngeal cancer for 1 dose alcohol (10g ethanol)/day [49]; ^6^ Adapted from [50]; ^7^ OR for head and neck cancer in never drinkers smoking > 3–5 cigarettes/day [51]; ^8^ One commercially available yogurt (approximately 150 mL)/day with lowest reported ACH level, consumed in 3 min. Adapted from [52]; ^9^ Three commercially available yogurts (approximately 3 × 150 mL)/day with highest reported ACH level, each consumed in 5 min. Adapted from [52]; ^10^ One commercially available apple/day with lowest reported ACH level, consumed in 3 min. Adapted from [52]; ^11^ Three commercially available apples/day with highest reported ACH level, each consumed in 5 min. Adapted from [52]. ?: no data available.

**Table 3:** Odds ratios (OR) and 95% confidence intervals (CI) for gastric and esophageal cancer in relation to selected food habits based on epidemiological surveys from high-risk areas.

Type of Cancer	Food Habit	OR	95%CI	*p*-Value
Esophageal	Pickled vegetables ^1^	2.10	1.47–3.0	<0.001
	*Mursik* milk ^2^	3.72	1.96–7.14	<0.001
Gastric	Pickled vegetables ^3^	1.52	1.37–1.68	<0.001
	Yogurt ^4^	16.26	2.1–125.7	0.008
	Cheese ^4^	15.05	1.6–137.0	0.01
	Moldy food ^4^	1.92	1.2–3.0	0.004
	Pickling liquid ^4^			
	Brine	4.76	2.2–10.4	<0.001
	Vinegar + brine	3.34	1.6–7.0	0.002

^1^ Data based on a large meta-analysis from China [138]; ^2^ Fermented milk used in western Kenya, an area with high incidence of esophageal cancer [56,139]. ORs have not been adjusted; ^3^ Data based on large meta-analysis including several different countries, such as China, Korea, and Japan [114]; ^4^ Food habits in high-risk area for gastric cancer in northeastern Iran with adjusted ORs [117].

**Table 4:** Environmental, genetic, disease-based, and iatrogenic risk factors or conditions for upper GI tract cancer and their effect on local acetaldehyde (ACH) exposure via saliva. Without the presence of ethanol or tobacco, salivary ACH levels are under the detection limit (<2 µM, [12,50]). The estimated concentration range of mutagenicity for ACH is 40–100 µM [20].

Environmental risk factors: ➢ Alcohol consumption: ▪ Instant exposure: Microbial formation of ACH from ethanol (up to > 260 µM) in saliva for 0–10 min after each sip of alcohol [13] ▪ Long-term exposure: Ethanol diffused to saliva from blood is oxidized mainly by oral microbes to ACH (mean 25 µM) in saliva for as long as ethanol remains in the human body [12] ➢ Tobacco smoking: ▪ Mean 260 µM of ACH in saliva for as long as smoking continues [50] ➢ Smoking + drinking: ▪ Seven-fold increase in local ACH exposure via saliva [50] ➢ Chronic smoking and heavy drinking: ▪ Both modify oral flora resulting in approximately 100% increase in salivary ACH production from ethanol both in vitro and in vivo [50,79] ➢ *Foodstuffs containing alcohol or ACH or both*: ▪ There is no evidence that ACH derived from ethanol or ACH-containing “non-alcoholic” beverages and food (or both) is less carcinogenic than ACH derived from official alcoholic beverages [7,8] Gene polymorphism-based risk conditions: ➢ ALDH2 deficiency among alcohol-consuming East Asians [37,40]: Mean two-fold additional ACH exposure via saliva and five-fold via gastric juice compared to ALDH2-active subjects for as long as alcohol stays in the human body [32,33] ➢ Highly active ADH among Caucasians [126]: In homozygotes, significantly higher salivary ACH levels following alcohol ingestion than in heterozygotes or in those with normal ADH [127] Disease-based risk conditions: ➢ Poor oral hygiene [145,146]: Associated with 100% higher in vitro ACH production in saliva from ethanol [95] ➢ *Helicobacter pylori* [115]: Produces ACH from ethanol at least in vitro [121,122] ➢ Atrophic gastritis [147]: Hypochlorhydric or achlorhydric stomach is colonized by oral microbes that produce ACH from ethanol in mutagenic concentrations [107,108,118] ➢ APECED (autoimmune polyendocrinopathy-candidiasis-ectodermal dystrophy): Rare disease characterized by chronic oral candidiasis and increased risk for oral cancer [84,88] Iatrogenic risk conditions: ➢ Long-term use of drugs suppressing gastric acid secretion (PPIs, H2-blockers) [111]: Microbial colonization of stomach results in enhanced local ACH production from ethanol [32,119]
